# Digitizing tuberculosis treatment monitoring in Wuhan city, China, 2020–2021: Impact on medication adherence

**DOI:** 10.3389/fpubh.2023.1033532

**Published:** 2023-03-01

**Authors:** Mengxian Zhang, Guiyang Wang, Hina Najmi, Aashifa Yaqoob, Tao Li, Yinyin Xia, Jianjun Ye, Shuangyi Hou, Ye Xiao, Liping Zhou, Yuehua Li

**Affiliations:** ^1^Institute for Tuberculosis Control and Prevention, Hubei Provincial Center for Disease Control and Prevention, Wuhan, Hubei, China; ^2^Wuhan Pulmonary Hospital, Wuhan Institute for Tuberculosis Control, Wuhan, Hubei, China; ^3^Health Services Academy, Islamabad, Pakistan; ^4^Common Management Unit (TB, HIV/AIDS & Malaria), Islamabad, Pakistan; ^5^University of Bergen, Bergen, Norway; ^6^National Center for Tuberculosis Control and Prevention, Chinese Center for Disease Control and Prevention, Beijing, China; ^7^Beijing SINOVO Power Technology LTD., Beijing, China

**Keywords:** tuberculosis, China, medication adherence and treatment outcome, digital adherence technology, operational research

## Abstract

**Introduction:**

Digital technologies can improve adherence to tuberculosis (TB) treatment. We studied the impact of digitizing TB treatment monitoring on adherence among TB patients in Wuhan, China, during 2020-2021.

**Methods:**

We compared an electronic system introduced to monitor TB medication adherence (e-Patient Service System; e-PSS) with the p paper-based standard of care (TB Control Information System; TCIS) in terms of prescribed TB treatment doses taken by patients and patient outcome after six months of follow up. We designed a cross sectional study using retrospective data for all drug susceptible pulmonary TB patients recorded on both systems. The main indicators were: compliant first follow up visit (within 3 days of start of treatment); medication adherence (80% or more of monthly doses taken); and end of treatment success ratio.

**Results:**

A total of 1,576 TB patients were recorded in TCIS in July September, 2020 and 1,145 TB cases were included in e-PSS in January March, 2021. The distribution of patient demographic and clinical features was similar between the two groups. A larger proportion from the e-PSS group visited the community doctor in the first three days compared with the TCIS group (48.91 versus 29. 76 % respectively). Medication adherence was also higher in the e-PSS group during the 6 months of treatment than in the TCIS group (84. 28 versus 80.3 3 % respectively). Treatment success was 92.52% in the e-PSS group and 92.07% in the TCIS group. Multivariate logistic regress ion analysis demonstrated that adjusted odds ratios for compliant first follow up visit, medication adherence and favorable treatment outcome in the e-PSS versus TCIS groups were 2.94 (95% 2.47 3.50), 1.33 (95% 1.08 1.63), and 1. 12 (95% CL: 0.79 1.57) respectively.

**Discussion:**

This study revealed improvements in TB care following an intervention to monitor treatment digitally in patients in Wuhan, China.

## Introduction

Tuberculosis (TB) is a communicable disease that remains a major global public health threat (1). In 2021, 6.4 million people were newly diagnosed with TB and an estimated 1.6 million deaths were reported, making TB the second most important infectious killer worldwide after COVID-19 that year (2). China is one of the 30 high-TB burden countries in the world, and the incidence rate as of 2021 is currently 55/100,000, with 780,000 new reported TB cases (2). Wuhan is the biggest city in China's central region, with 5,596 new reported TB cases in 2021.

Most TB treatment regimens in use globally require multiple drugs to be taken daily for 6 months in order to achieve high efficacy. This long treatment is one of the most significant obstacles to TB control and interruption can lead to the emergence of drug-resistant TB (DR-TB) and continued spread. In a patient-level pooled analysis of treatment-shortening regimens for drug-susceptible pulmonary tuberculosis, non-adherence was the most significant risk factor for an unfavorable outcome: Missing 10% of doses or more was associated with an adjusted hazard ratio of 5.7 [95% confidence interval (CI): 3.3–9.9] (3). Therefore, many programs support patients with TB to improve adherence and completion of treatment.

Different strategies are recommended by the World Health Organization (WHO) to help people with TB take their treatment using person-centered approaches (4). These include in-person assistance and the use of different digital adherence technologies. A systematic review found that ~52% of patients with TB adopted self-administered therapy (SAT) in China, attesting to the difficulties in implementing in-person support in large high-TB burden countries (5). Compared with directly observed therapy (DOT) alone, SAT has been associated with lower rates of treatment success, adherence, and sputum smear conversion as well as higher rates of development of drug resistance (6, 7). China's National TB Programme (NTP) offers different forms of treatment adherence support, such as home visits or phone calls from community or village doctors (village-level licensed general practitioners), family members, or volunteers. The village doctors are expected to visit each patient every 10 days during the intensive phase followed by once a month in the continuation phase. Since village supporters may be busy with their own routine business and may find it difficult to remind and follow patients for such a long period, digital technologies are being increasingly used for improving the adherence of patients with TB.

Several digital technologies are being applied to help TB treatment completion globally (7), such as short message services (SMSs) *via* mobile phones, phone calls, and video-supported therapy (VOT) (4, 8). In general, these digital technologies may not have a clear impact on treatment success compared with in-person observation, while they help save resources and improve adherence (9, 10). Evidence in Northwest Ethiopia reported that mobile phone-based weekly refilling with a daily medication reminder system improved adherence to patient-centered TB treatment (79 vs. 66.4%; RR = 1.63, 95% lower CI 1.16, one-tailed, *p* = 0.02); however, there was no significant effect on treatment success (89.5 vs. 85.1%, *p* = 0.12) (11). Similar evidence from a systematic review showed that clinic attendance and TB treatment completion were higher in people receiving pre-appointment reminder phone calls [clinic attendance: 66 vs. 50%, relative risk (RR) = 1.32, 95% CI 1.10–1.59; TB treatment completion: 100 vs. 88%, RR = 1.14, 95% CI 1.02–1.27] (12). Studies show that VOT achieved adherence to anti-TB treatment and significantly reduced the time and money patients spent on their treatment (13, 14). In China, studies showed that implementing an electronic medication monitor improved the treatment success rate (15) and medication adherence (16). For example, a pill box could remind the patient to take medicine on time and record the patient's medication each time the patient opens the device (16). Innovative approaches that can aid healthcare workers in tracing patients and supporting patients in adhering to TB treatment are needed in China.

Very few studies have evaluated the impact of data management on TB treatment outcomes. In this study, we looked at the impact of electronic monitoring of TB medication adherence (e-Patient Service System; e-PSS) on prescribed care and TB treatment completion 6 months after the start in Wuhan, China, in 2021. We compared this with a paper-based standard of care (TB Control Information System; TCIS) in late 2020. We used the findings to discuss the implications for programmatic enhancements.

## Methods

### Study design and setting

This was a comparative cross-sectional study using retrospective data to compare between TCIS and e-PSS in Wuhan, China. With a population of more than 11 million people, Wuhan is the capital city of Hubei province in central China. The city has an area of approximately 8,569.15 square kilometers and consists of 17 districts with TB programs (17).

In China, the Tuberculosis Control and Prevention Strategy provides patient-centered “prevention, diagnosis, treatment, management, and education” as part of comprehensive care services, aimed at reducing TB incidence and death and the economic burden on patients. Alongside the strategy, TB surveillance has been strengthened in China in recent years through an Infectious Disease Reporting System and Tuberculosis Information Management System (18). Through these two systems, both TB notification and TB treatment outcomes can be monitored in a timely manner (19). These measures help optimize TB service provision systems and improve the quality of case detection and treatment management. However, there is no national system to manage and monitor medication adherence and follow-up on an individual patient level. China is looking to establish a technical support system to assist in TB patient treatment management. At this time, e-PSS, developed by Beijing SINOVO Power Technology Company Limited, has been recently introduced in Chinese primary health institutions, in Beijing (20) and Tianjin (21). It is targeted mainly for use by hospitals, community clinics, and patients with TB based on the Technical Guide for Tuberculosis Control in China. Early results show that these tools help to combine patient data from hospitals and communities and lead to improvements in management and services for both patients and medical staff. To maintain the operation of this system, the Institution for Tuberculosis Control and Prevention will regularly supervise and inspect the hospital and community clinics.

**Figure 1:**
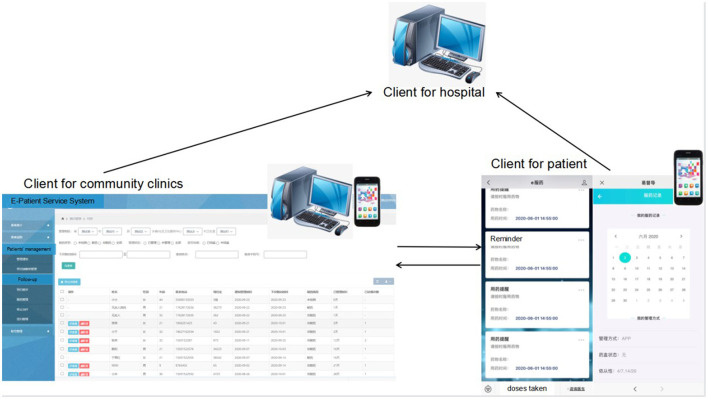
E-Patient Service System.

In Wuhan, both TCIS and e-PSS have been introduced to improve adherence and treatment outcomes among patients with TB. TCIS was the paper-based standard of care, used by community doctors. The Wuhan Institute for Tuberculosis Control established TCIS with a paper-based standard in 2008. The functions of TCIS are recording diagnosis and treatment status, collecting medication adherence records, and recording routine community home visits. E-PSS (Figure 1) was introduced by the Wuhan Institute for Tuberculosis Control in 2021, as a new TB patient treatment support system administered through a website, mobile phone application, or WeChat (an instant messaging service). The e-PSS has four modules: patient referral to the TB hospital, tracing of patients in the community, patient treatment management, and health education. The patient management module can (1) assist community clinic doctors to manage and follow up patients with TB *via* mobile phone, (2) send reminder alerts of medication and follow-up visits to community doctors and patients, and (3) allow hospital doctors to get feedback from patients and monitor patients' medication status in real time. In our study, we considered TCIS to manage patients with TB diagnosed before 2021 and e-PSS to manage patients with TB diagnosed after 2020.

### Ethics approval

Permission for the study was sought from Hubei Provincial Center for Disease Control and Prevention and Wuhan Pulmonary Hospital, Wuhan, China. As this study involved analysis of routinely collected anonymous secondary data, the ethics committee waived the need for informed consent and formal ethics approval.

### Study population

The study population included all patients with drug-sensitive pulmonary TB registered with TCIS and e-PSS residing in Wuhan, China. The study included all 17 districts in Wuhan with a TB program.

### Indicators, sources of data, and data collection

The three indicators used to compare the performance of TCIS and e-PSS were the following: the proportion of patients in whom there was a first follow-up visit to the community doctor within 3 days of the start of treatment; medication adherence defined as 80% or more of prescribed monthly doses taken; and the percentage of end-of-treatment success in patients under e-PSS and TCIS.

All data were extracted from TCIS and e-PSS. The data for the e-PSS group were collected from January to March 2021, and the data for the TCIS group were retrospectively collected from July to September 2020. All records were consolidated into a single database in Excel with standardized fields.

### Analysis plan

Descriptive statistics were used to describe the characteristics of the study population, using means to summarize continuous variables and percentages for categorical variables. The chi-square test was used to analyze categorical variables. Multivariate logistic regression analysis was also carried out to adjust for confounding factors. A *p*-value < 0.05 was considered statistically significant. All statistical analyses were conducted using SPSS version 25.0. The forest plots were created using the forest plot package for R software version 4.1.1.

## Results

A total of 1,576 patients with TB were recorded in TCIS between July and September 2020 in Wuhan, China, and 1,145 TB cases were included in e-PSS during the period from January to March 2021. Of these, the age group of 45–64 years was the largest in both systems, 32.49 and 33.36%, respectively, and the majority of cases were men (62.56 and 66.90%, respectively). The proportion of unemployed was higher in TCIS (37.82%) than in e-PSS (24.54%), whereas employed (22.14%) was lower than in e-PSS (26.64%). The majority of the participants were newly diagnosed patients with TB (95.24% in TCIS and 94.85% in e-PSS), and more than 49% were laboratory bacteriological positive. There were nearly 85% of cases without previous history and ~7% with diabetes (Table 1).

**Table 1:** Patient demographics and clinical characteristics recording in TCIS and e-PSS during 2020–2021 in Wuhan.

**Variables**	**TCIS** **(*n* = 1,576)** ***N* (%)**	**E-PSS** **(*N* = 1,145)** ***N* (%)**	***P*-value**
Age (yr)			0.324
0–14	14 (0.89)	6 (0.52)	
15–24	184 (11.68)	109 (9.52)	
25–44	501 (31.79)	369 (32.23)	
45–64	512 (32.49)	382 (33.36)	
>65	365 (23.16)	279 (24.37)	
Mean (SD)	47.89 (19.27)	48.92 (18.66)	
Gender			0.017
Male	986 (62.56)	766 (66.90)	
Female	590 (37.44)	379 (33.10)	
Occupation			< 0.001
Farmer	166 (10.53)	144 (12.58)	
Employee	349 (22.14)	305 (26.64)	
Unemployed	596 (37.82)	281 (24.54)	
Retiree	234 (14.85)	239 (20.87)	
Others	231 (14.66)	176 (15.37)	
Type of TB			0.639
New case	1,501 (95.24)	1,086 (94.85)	
Previously treated	75 (4.76)	59 (5.15)	
Category of TB report			0.004
Positive	781 (49.56)	647 (56.51)	
Negative	742 (47.08)	461 (40.26)	
TB pleurisy	44 (2.79)	31 (2.71)	
Unknown	9 (0.57)	6 (0.52)	
Previous history			0.381
None	1,329 (84.33)	978 (85.41)	
Diabetes	114 (7.23)	91 (7.95)	
Silicosis	1 (0.06)	0 (0.00)	
Mental disease	2 (0.13)	2 (0.17)	
Other	130(8.25)	74 (6.46)	

**Figure 2:**
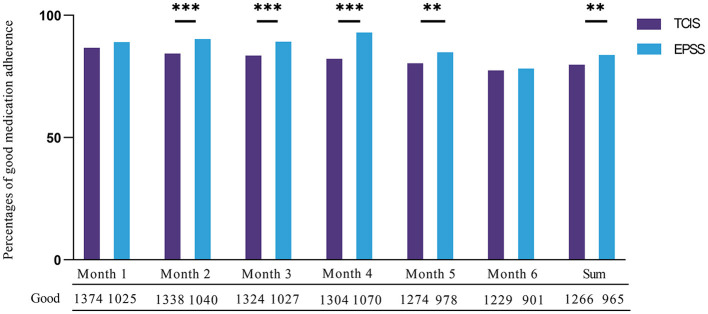
Medication adherence recorded in TCIS and e-PSS for 6 months. Good medication adherence defined as more than 80% of prescribed monthly doses taken. **P < 0.01. ***P < 0.001. All the bar graphs were grouped by month; the bar graph on the left in each group depicted the data distribution of good medication adherence in TCIS, and the number of good medication adherence was denoted by the digit under each column. Conversely, the bar graph on the right depicted that in e-PSS.

During 6 months of follow-up of 1,576 patients in TCIS and 1,145 patients in e-PSS, there was a 19.15% increase in community doctors who visited patients within the first 3 days (from 29.76% in TCIS to 48.91% in e-PSS) (Table 2). There was a slightly lower percentage of patient months with good medication adherence (i.e., >80% of prescribed treatment doses taken) in the TCIS group compared with the e-PSS group (80.33 vs. 84.28%, respectively). Good medication adherence was higher in the e-PSS group than in the TCIS group for each of the 6 months of treatment (Figure 2).

**Table 2:** Status of community visit in TCIS and e-PSS during 2020–2021 in Wuhan, China.

**Variables**	**TCIS (*n* = 1,576)**	**E-PSS (*n* = 1,145)**	**Percentage difference (%)**	***P*-value**
Compliant first follow-up visit (based on days)				< 0.001
< 4	469 (29.76)	560 (48.91)	19.15	
4–14	380 (24.11)	286 (24.98)	0.87	
>14	706 (44.80)	200 (17.47)	−27.33	
Not evaluated	21 (1.33)	99 (8.65)	7.31	

After 6-month follow-up, 555 cases (35.22%) in TCIS and 169 cases (14.76%) in e-PSS were not evaluated. Using cases with known outcome status as the denominator, the percentages of favorable treatment outcomes (including cured and treatment completed) were 92.52% in the e-PSS group and 92.07% in the TCIS group (*P* = 0.704). A total of 41.92% were cured whereas 50.15% were categorized as treatment completed in TCIS as compared to e-PSS where 38.42% were cured and 54.10% were treatment completed. The proportions of unfavorable outcomes (including failure, death, lost to follow-up, transferred to RR/MDR-TB, and others) were 7.93 and 7.48% in TCIS and e-PSS, respectively (Table 3).

**Table 3:** Treatment outcomes among patients with TB recorded in TCIS and e-PSS during 2020–2021 in Wuhan, China.

Treatment outcome^*^	**TCIS (*n* = 1,021)**	**E-PSS (*n* = 976)**	***P*-value**
	***N*** **(%)**	***N*** **(%)**	
Favorable outcome	940 (92.07)	903 (92.52)	0.704
Cured	428 (41.92)	375 (38.42)	
Treatment completed	512 (50.15)	528 (54.10)	
Unfavorable outcome	81 (7.93)	73 (7.48)	
Failure	1 (0.10)	2 (0.20)	
Died	19 (1.86)	27 (2.77)	
Lost to follow up	9 (0.88)	4 (0.41)	
Transferred to RR/MDR-TB	8 (0.78)	2 (0.20)	
Other	44 (4.31)	38 (3.89)	

* There were 555 cases in TCIS and 169 cases in e-PSS without evaluation recorded.

**Figure 3:**
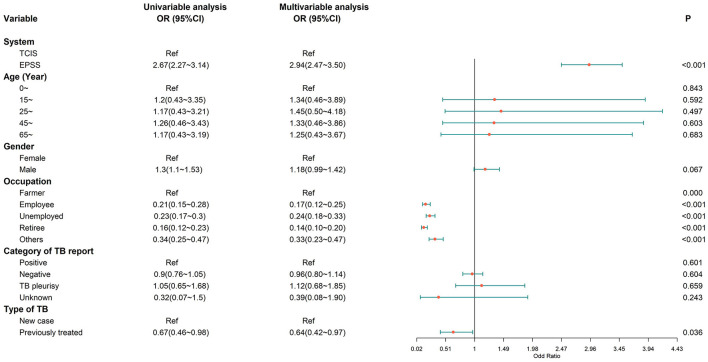
Multivariate logistic regression analysis on the factors associated with a compliant first follow-up visit. A compliant first follow-up visit, the first visit to the community doctor within 3 days of the start of treatment; OR, odds ratio; CI, confidence interval; model was adjusted for age, gender, occupation, category of TB report, and type of TB.

**Figure 4:**
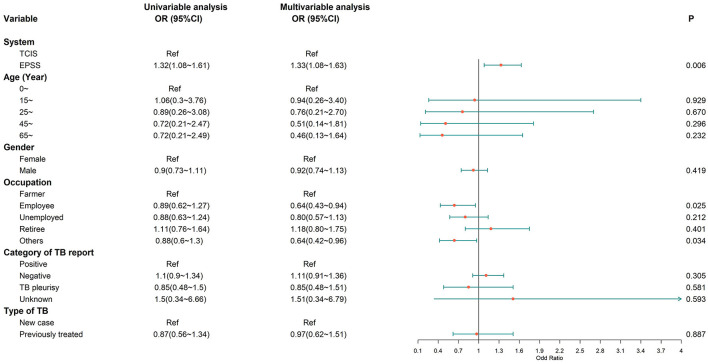
Multivariate logistic regression analysis on the factors associated with good medication adherence. Good medication adherence, more than 80% doses taken during 6 months; OR, odds ratio; CI, confidence interval; model was adjusted for age, gender, occupation, category of TB report, and type of TB.

**Figure 5:**
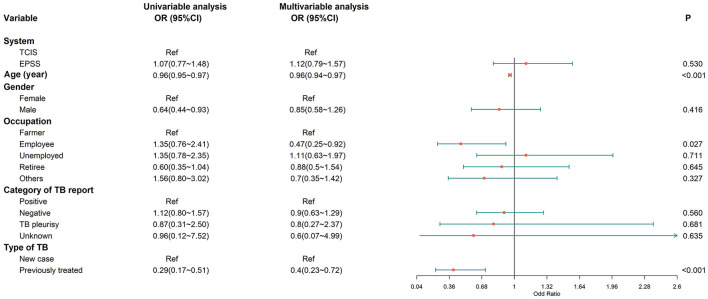
Multivariate logistic regression analysis on the factors associated with favorable treatment outcomes. Favorable treatment outcome, cured or treatment completed; OR, odds ratio; CI, confidence interval; model was adjusted for age, gender, occupation, category of TB report, and type of TB.

We retrospectively analyzed the association between independent variables (e-PSS/TCIS) and dependent variables (compliant first follow-up visit, good medication adherence, and favorable treatment outcome), after adjusting for covariates (age, sex, occupation, category of TB report, and type of TB). When comparing the e-PSS group with the TCIS group, a statistically significant association was observed with compliant first follow-up visit [adjusted OR: 2.94, (95% CI: 2.47–3.50); Figure 3] and good medication adherence [adjusted OR: 1.33, (1.08–1.63); Figure 4], while the association with favorable treatment outcome was not statistically significant [adjusted OR: 1.12 (0.79–1.57); Figure 5]. Patients with TB who had been previously treated were less likely to attend the first follow-up visit [adjusted OR: 0.64, (0.42–0.97)] and to have a favorable outcome (adjusted OR: 0.40, 95% CI: 0.23–0.72) than previously untreated patients. There was a 4% decrease in the odds of a favorable outcome (OR: 0.96, 95% CI: 0.94–0.97) for every 1-year increment in age.

## Discussion

Our study showed that the use of e-PSS among patients with TB could support compliant first follow-up visits and enhance medication adherence levels compared with TCIS during the project period (2020–2021) in Wuhan, China.

The e-PSS played an important role in tracing and managing patients with TB in the community. A compliant first follow-up visit was more frequent in e-PSS compared to TCIS. In e-PSS, community doctors can receive patients' information in a timely fashion *via* short message service (SMS) and complete all the work of follow-up visits with a smartphone and computer. However, community doctors only used paper files to record follow-up information in TCIS and lack of reminders.

The results demonstrated that e-PSS (84.28%) improved medication adherence among patients with TB as compared with TCIS (80.33%) during the 6 months of TB treatment. For the medication adherence data in TCIS, the community doctors asked the patients about the status of missed or delayed medication for the past 10 days or 1 month through face-to-face or telephone interviews. Through the e-PSS, patients with TB could record their medication adherence daily by mobile phone, and the community doctors could check the medication data of all patients instantly. There is an intervention of daily medication reminders by sending SMS in real time, which can greatly improve patient medication adherence. A study available from Northwest Ethiopia reported that mobile phone-based weekly refilling with a daily medication reminder system improved adherence to patient-centered TB treatment and provider–patient relationship (11). Thus, good adherence is significantly associated with increased use of adherence strategies and digital interventions. Meanwhile, e-PSS is convenient for patients with TB to view or search for authoritative and practical knowledge, such as medical guidance on the diagnosis. It also supports bi-directional communication between patients with TB and doctors when adverse drug reactions and concerns arose during the treatment course by using mobile phone applications directly. Similar evidence from Thailand, the CARE-call system, a mobile-based system, was able to prevent non-adherence in this rural setting and was satisfied by participants (22).

A systematic review of digital interventions, including short message service (SMS), video-observed therapy (VOT), and medication monitors (MMs), to support treatment for active TB showed that the effect of digital technologies to improve TB care remains limited (10). Another study in Karnataka, India, showed that using daily treatment regimens with an innovative adherence support tool among HIV-infected TB did not improve successful treatment outcomes (23). We could not find a statistically significant association with favorable treatment outcomes, which might be due to incomplete data on treatment outcomes, and such an association should be further explored in similar future studies of this type.

These findings are important not only for better medication adherence and successful treatment outcome among patients with TB but also for preventing TB to spread in public. There were several limitations of this study. First, when the study could not assess all patients' treatment outcomes, both systems mainly facilitate patient management, and there are no strict requirements for the entry of treatment outcomes; however, the result shows that the data of treatment outcomes in e-PSS are more complete than in TCIS. Second, our study design cannot compare two system effects at the same time, since TCIS stopped running in 2021. Third, as of now, e-PSS has only been running for more than a year, and the doctors are not skilled in operation, thus, the functions of the new system cannot be fully demonstrated. But what is certain is that the efficacy of the new system will become more and more high quality over time. Finally, because hospital follow-up function in e-PSS was not yet available in Wuhan, the data of hospital follow-up have not been involved in this study, and further operational research will be needed.

To the best of our knowledge, this is the first study that evaluates how digitizing data management influences TB medication adherence and patient outcomes. The digital approach we describe from Wuhan, China, in 2020–2021 improved follow-up visit attendance and medication adherence. We showed that the monitoring of TB treatment in the community using mobiles and cloud-based computing is feasible, and, in contrast to the current paper-based system, allows for access to real-time information and more timely action. We propose that such a system be more widely deployed and its effect on TB treatment efficiency and effectiveness be studied further.

## Data availability statement

The datasets presented in this article are not readily available because we need to ensure privacy of Chinese citizens' information is under protection. Requests to access the datasets should be directed to zhangmengxian1990@163.com.

## Ethics statement

Written informed consent was obtained from the minor(s)' legal guardian/next of kin for the publication of any potentially identifiable images or data included in this article.

## Author contributions

MZ and GW designed and implementation of the research, completed the analysis, and drafted the manuscript. HN and AY provided expertise with the data and edited the manuscript. TL and YiX provided guidance with regards to theory. YeX provided raw data from the system. JY, SH, LZ, and YL supervised the project, critically reviewed, and approved the final version of the manuscript.
