# Corrigendum: Digitizing tuberculosis treatment monitoring in Wuhan city, China, 2020–2021: Impact on medication adherence

**DOI:** 10.3389/fpubh.2023.1191444

**Published:** 2023-04-04

**Authors:** Mengxian Zhang, Guiyang Wang, Hina Najmi, Aashifa Yaqoob, Tao Li, Yinyin Xia, Jianjun Ye, Shuangyi Hou, Ye Xiao, Liping Zhou, Yuehua Li

**Affiliations:** ^1^Institute for Tuberculosis Control and Prevention, Hubei Provincial Center for Disease Control and Prevention, Wuhan, Hubei, China; ^2^Wuhan Pulmonary Hospital, Wuhan Institute for Tuberculosis Control, Wuhan, Hubei, China; ^3^Health Services Academy, Islamabad, Pakistan; ^4^Common Management Unit (TB, HIV/AIDS & Malaria), Islamabad, Pakistan; ^5^University of Bergen, Bergen, Norway; ^6^National Center for Tuberculosis Control and Prevention, Chinese Center for Disease Control and Prevention, Beijing, China; ^7^Beijing SINOVO Power Technology LTD., Beijing, China

**Keywords:** tuberculosis, China, medication adherence and treatment outcome, digital adherence technology, operational research

In the published article, an author name was incorrectly written as “Linping Zhou”. The correct spelling is “Liping Zhou.”

We have also checked and ensure that the initials used in the Author contributions section or elsewhere in the article are correct.

The authors apologize for this error and state that this does not change the scientific conclusions of the article in any way. The original article has been updated.

